# Hypoxia Aggravates Inhibition of Alveolar Epithelial Na-Transport by Lipopolysaccharide-Stimulation of Alveolar Macrophages

**DOI:** 10.3390/ijms23158315

**Published:** 2022-07-27

**Authors:** Emel Baloglu, Kalpana Velineni, Ezgi Ermis-Kaya, Heimo Mairbäurl

**Affiliations:** 1 Department of Medical Pharmacology, School of Medicine, Acibadem Mehmet Ali Aydinlar University, 34752 Istanbul, Turkey; emel.baloglu@acibadem.edu.tr; 2 Translational Lung Research Center Heidelberg (TLRC-H), Part of the German Center for Lung Research (DZL), 69120 Heidelberg, Germany; kalpu8757@gmail.com (K.V.); ezgiermis@gmail.com (E.E.-K.); 3 Medical Clinic VII, Sports Medicine, University Hospital Heidelberg, 69120 Heidelberg, Germany; 4 Translational Pneumology, University Hospital Heidelberg, 69120 Heidelberg, Germany

**Keywords:** alveolar epithelial Na-channels, Na/K-ATPase, pulmonary edema, inflammation, iNOS, hypoxia, alveolar macrophages

## Abstract

Inflammation and hypoxia impair alveolar barrier tightness, inhibit Na- and fluid reabsorption, and cause edema. We tested whether stimulated alveolar macrophages affect alveolar Na-transport and whether hypoxia aggravates the effects of inflammation, and tested for involved signaling pathways. Primary rat alveolar type II cells (rA2) were co-cultured with rat alveolar macrophages (NR8383) or treated with NR8383-conditioned media after stimulation with lipopolysaccharide (LPS; 1 µg/mL) and exposed to normoxia and hypoxia (1.5% O_2_). LPS caused a fast, transient increase in TNFα and IL-6 mRNA in macrophages and a sustained increase in inducible nitric oxide synthase (NOS2) mRNA in macrophages and in rA2 cells resulting in elevated nitrite levels and secretion of TNF-α and IL-6 into culture media. In normoxia, 24 h of LPS treated NR8383 decreased the transepithelial electrical resistance (TEER) of co-cultures, of amiloride-sensitive short circuit current (ISC_Δamil_); whereas Na/K-ATPase activity was not affected. Inhibition was also seen with conditioned media from LPS-stimulated NR8383 on rA2, but was less pronounced after dialysis to remove small molecules and nitrite. The effect of LPS-stimulated macrophages on TEER and Na-transport was fully prevented by the iNOS-inhibitor L-NMMA applied to co-cultures and to rA2 mono-cultures. Hypoxia in combination with LPS-stimulated NR8383 totally abolished TEER and ISCΔamil. These results indicate that the LPS-stimulation of alveolar macrophages impairs alveolar epithelial Na-transport by NO-dependent mechanisms, where part of the NO is produced by rA2 induced by signals from LPS stimulated alveolar macrophages.

## 1. Introduction

Lung injury is often caused by particles, bacteria and viruses entering terminal airways and may have detrimental effects if not treated. Alveolar macrophages play a central role in maintaining lung homeostasis and protection of the alveolar epithelial function and integrity by increased formation of inflammatory mediators, reactive oxygen and nitrite species, a variety of other signaling molecules, and recruiting neutrophils [1] to the inflammation site. LPS, by signaling via Toll-like receptor 4 (TLR4) and p38 (MAPK) and STAT-3 [2], induces the release of pro-inflammatory cytokines such as TNF-α, IL-1β, IL-6, and IL-8 from lung resident alveolar macrophages [3], which stimulate the expression of the inducible nitric oxide synthase (iNOS), as well as NFkB, which further modulates cytokine production [4]. At the same time nitric oxide (NO) and superoxide dependent peroxynitrite play an important role in mediating NO-related lung epithelial injury [5]. While these factors may exacerbate lung damage, alveolar macrophages are also the site of producing anti-inflammatory factors such as IL-10 and GM-CSF [6], which are crucial for resolving the inflammation and recruiting other immune cells to the sites of inflammation. Despite this protective role, however, inflammatory signals released by macrophages also induce leaks of the alveolar wall and impair alveolar Na-reabsorption, which can lead to alveolar edema [7].

Alveolar edema impairs oxygen diffusion into blood and limits oxygen supply to the alveolar epithelium causing cellular hypoxia resulting in inefficient alveolar fluid clearance [8,9,10]. Hypoxia also increases the permeability of the alveolar barrier [11,12] and activates alveolar macrophages [13]. Together, inflammation and hypoxia might aggravate edema formation and lung damage, and might even contribute to systemic inflammation and sepsis.

A major function of alveolar epithelial cells (A2) is to clear excess alveolar fluid by active Na^+^ transport, where apical amiloride-sensitive epithelial Na^+^ channels (ENaC) and basolateral Na/K-ATPase mediate Na-entry and extrusion, respectively. This generates the osmotic driving force for the removal of excess water from the alveolar surface [14]. Both Na-transport mechanisms are decreased when the alveolar epithelium becomes hypoxic [15,16,17,18]. Similarly, the impairment of Na-transport is common in acute lung injury [19] and acute respiratory distress syndrome (ARDS) [20] due to viral and bacterial pneumonia. The reduced alveolar fluid clearance in ARDS correlates with high mortality rates [21]. In addition to detrimental effects of stimulated macrophages on alveolar fluid homeostasis, A2 cells also contribute to lung inflammation by releasing inflammatory cytokines [22] and modulate the function of the inflammatory cells by secreting IL-6, MCP-1, and GM-CSF [23]. To date some in-vitro studies focused on the individual effects of the macrophages and A2 cells in response to inflammatory signals and hypoxia. However, the relative contribution of inflammatory signals from macrophages and alveolar epithelium, and the additional effect of hypoxia on alveolar reabsorption is not well defined.

The major goal of this study was to elucidate intracellular signaling that leads to the inhibition of alveolar epithelial Na transport by endotoxin-stimulated macrophages in combination with hypoxia. We show here different in-vitro models that study the interaction of rat alveolar macrophages with primary rat alveolar epithelial cells (rA2) so that the pro-inflammatory response of macrophages propagates to the rA2 cells resulting in alveolar epithelial NO-dependent inhibition of Na-transport and barrier function. Results also indicate that hypoxia dominates and aggravates the effect of lipopolysaccharide (LPS) and macrophage-induced inhibition of transport.

## 2. Results

### 2.1. Effects of LPS Stimulated Macrophages on Ion-Transport of rA2 Cells

**Figure 1:**
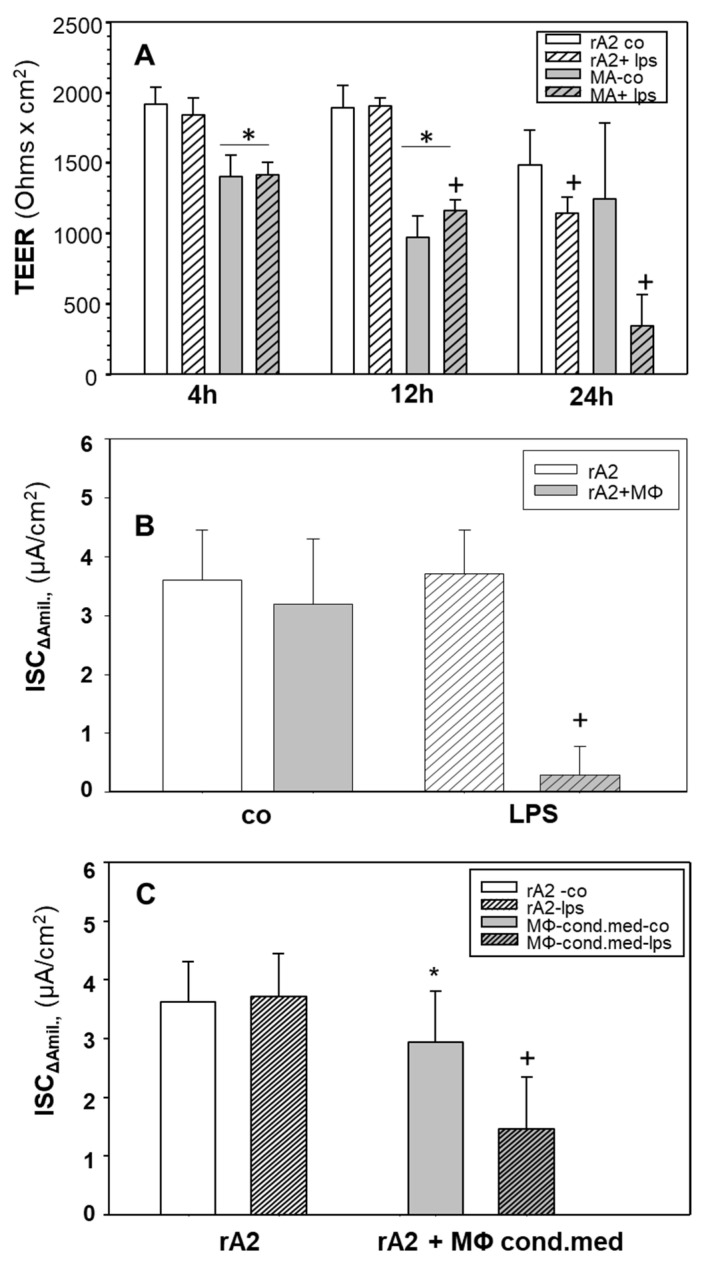
Effects of LPS on the transepithelial resistance and ion transport of rA2 cells in mono- and co-culture with alveolar macrophages and of conditioned medium obtained from LPS stimulated macrophages. Mono and co-cultures of rA2 cells with alveolar macrophages were prepared as described in the Methods section and treated with LPS (1 μg/mL) at indicated time points. Transepithelial electrical resistance (TEER) was measured by EVOM (Ohms × cm^2^) (**A**). Short circuit currents were measured in Ussing chambers in mono and co-cultures treated with LPS (1 μg/mL) for 24 h (**B**). Conditioned macrophage media was prepared by exposing mono-cultures of macrophages to LPS (1 µg/mL) for 24 h and kept on rA2 monolayers for 24 h. ENaC activity was measured as the amiloride (10 µM) sensitive component of I*eq* in Ussing chambers (ISC_Δamil_). (**C**). Mean values ± SD of 5–11 experiments from at least 3 independent preparations of primary rA2 cells. * *p* < 0.001 effect of culturing macrophages or conditioned media with rA2 cells, + *p* < 0.001 effect of LPS treatment compared to relevant controls. MΦ: rat alveolar macrophages; rA2: rat alveolar epithelial cells alone; rA2 co: control rat alveolar epithelial cells that did not receive MΦ, MΦ conditioned medium or LPS; MA: MΦ and rA2 co-culture.

Figure 1A shows that applying LPS directly on rA2 cell monolayers for 24 h decreased the transepithelial electrical resistance (TEER; *p* < 0.05); shorter exposure had no effect. Co-culture for 4 and 12 h but not 24 h with unstimulated macrophages decreased the TEER of rA2 cells compared to control monolayers (*p* < 0.001). The addition of LPS to macrophage-rA2 co-cultures for 24 h further decreased (~75%) TEER compared to control (*p* = 0.001), whereas LPS treatment of rA2 cell mono-layers decreased TEER by only ~20% (*p* = 0.05). In further experiments, only 24-h exposures are shown.

The presence of non-stimulated macrophages had no effect on the amiloride-sensitive short circuit current (ISC_Δamil_), which is a measure of epithelial Na Channel (ENaC) activity, compared with rA2 mono-layers (*p* = 0.173; Figure 1B). LPS stimulation of macrophages in co-culture with rA2 cells significantly decreased ISC_Δamil_ (*p* < 0.001) compared with non-stimulated co-cultures. Co-culture with non-stimulated and LPS-stimulated macrophages did not affect ISC_ampho_ indicating that Na/K-ATPase function was not impaired (data not shown).

We also tested whether the side of application of MΦ plus LPS to A2 cells, on apical or basolateral, affected the response. Supplementary Figure S1 shows that MΦ seeded apically on rA2 cells slightly decreased amiloride-sensitive short circuit current (ISC_Δamil_) compared with the rA2 mono-layer (*p* = 0.018), whereas basolateral seeding had no effect. In both types of co-cultures, apical and basolateral LPS application decreased ISC_Δamil_ compared to respective controls (*p* < 0.001). There was no difference in ISC_Δamil_ between apical and basolateral application of LPS in the co-cultures (*p* = 0.279). For further experiments we used co-cultures of basolaterally seeded MΦs to better distinguish the role of any signaling molecules releasing from individual cells. This also allowed us to collect material for other measurements without contamination from the two cell types, which apical co-cultures may cause.

We also used conditioned media from MΦ to test whether the close vicinity of MΦ is required to exert effects on rA2 Na-transport. Figure 1C shows that not only stimulated macrophages but also 24-h incubation with conditioned media from LPS-stimulated macrophages inhibited ISC_Δamil_ transport by ~50% (*p* < 0.001). Conditioned medium from control macrophages slightly decreased ISC_Δamil_ (*p* = 0.034).

### 2.2. Cytokines, iNOS, NO/Nitrite

**Figure 2:**
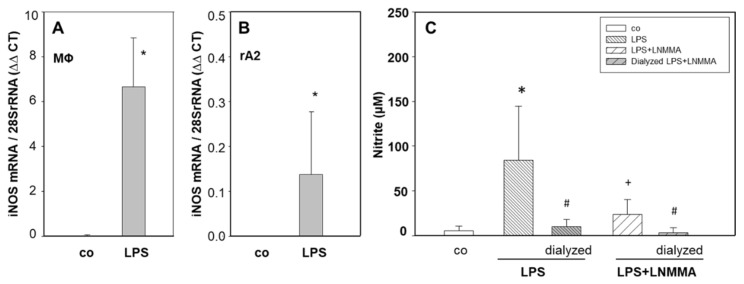
Effect of LPS treatment on the mRNA expression of iNOS in macrophages and rA2 mono-layers and nitrite levels in dialyzed macrophage conditioned media. Macrophage (**A**) and rA2 cells (**B**) were treated with LPS (1 µg/mL) for 24 h. mRNA expression was normalized to 28S-rRNA. Mean values ± SD of 4 to 5 independent cell preparations. Conditioned macrophage media were prepared by exposing mono-cultures of macrophages to LPS (1 µg/mL) and L-NMMA (1 mM) for 24 h (**C**). Five ml of conditioned medium was dialyzed and nitrite production was measured. Mean values ± SD of 3–6 independent preparations. Level of significance was *p* < 0.05: * effect of LPS treatment, # effect of dialysis, + effect of L-NMMA treatment. MΦ: rat alveolar macrophages; rA2: rat alveolar epithelial cells.

The treatment of macrophage- and rA2-mono-cultures with LPS for 24 h resulted in the typical upregulation of mRNA (Supplementary Figure S2A–D) and protein of TNFα and IL-6 (Supplementary Figure S3A,B). Stimulation of expression was much more pronounced in macrophages than in rA2 cells (Supplementary Figure S2A,B). Figure 2A,B show that LPS treatment increased iNOS mRNA levels in mono-cultures of macrophages and of rA2 cells. Supplementary Figure S4 shows that treatment with LPS increased nitrite levels in culture media from mono-cultures of rA2 cells and macrophages in a time-dependent manner indicating increased NO-production. After 24 h, levels were approximately 4-fold higher in media from macrophages than from rA2 cells. These results show that macrophages are more sensitive to producing proinflammatory cytokines and nitrite upon LPS treatment than rA2 cells.

**Figure 3:**
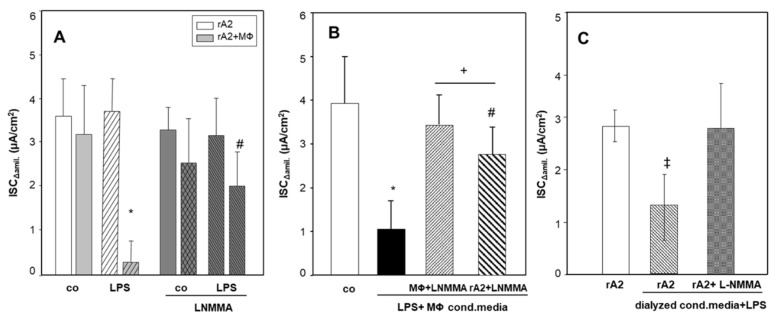
Effect of inhibiting of iNOS activity with LNMMA on ion transport activity in co-cultures and cells exposed to LPS stimulated conditioned media. Mono and co-cultures of rA2 cells with alveolar macrophages were pretreated with L-NMMA (1 mM) for 30 min prior to LPS (1 μg/mL) stimulation. ENaC activity was measured as the amiloride (10 µM) sensitive component of I*eq* in Ussing chambers (ISC_Δamil_) (**A**). Conditioned macrophage media from mono-cultures of macrophages treated with LPS (1 µg/mL) and L-NMMA (1 mM) was kept on rA2 cell monolayers and on macrophages for 24 h. (**B**). Conditioned macrophage media from mono-cultures of macrophages treated with LPS (1 µg/mL) were dialyzed and kept on rA2 monolayers for 24 h, in parallel rA2 cells treated with L-NMMA (1 mM) 30 min prior receiving LPS treated conditioned media (**C**). Mean values ± SD of 4–6 experiments from at least 3 independent preparations of primary rA2 cells. * effect of LPS treatment of co-cultures and conditioned media *p* < 0.001; + effect of L-NMMA, # effect of L-NMMA treatment of rA2 cells compared to MΦ + L-NMMA, ‡ effect of dialysis *p* < 0.001. MΦ: rat alveolar macrophages; rA2: rat alveolar epithelial cells.

We aimed to test whether NO or other factors induced by LPS-stimulation of macrophages caused the inhibitory effects on rA2 Na-transport. Figure 2C shows that nitrite levels in conditioned media from LPS-stimulated macrophages could be removed nearly completely by dialysis (*p* = 0.029). Dialyzing LPS treated macrophage conditioned media did not affect TNF-α and IL-6 levels (Supplementary Figure S3A,B, respectively). Inhibition of inducible nitric oxide synthase (iNOS) with L-NMMA prevented the increase in nitrite levels in LPS-treated macrophages (*p* = 0.035; Figure 2C). Figure 3A shows that inhibiting iNOS with L-NMMA prevented the inhibition of ISC_ΔAmil_ caused by LPS stimulated macrophages in co-cultures (*p* = 0.009), whereas L-NMMA had no effect on control rA2 cells (*p* = 0.284). Figure 3B shows that inhibition (−75%) of ISC_Δamil_ was almost completely prevented, when rA2 cells were incubated for 24 h with conditioned media from LPS- and L-NMMA treated macrophages (*p* = 0.01). Interestingly, most of the decrease in ISC_Δamil_ was also prevented in rA2 cells exposed to conditioned media from LPS-treated macrophages, when L-NMMA was added to the rA2 cells prior receiving the conditioned media (*p* = 0.013, Figure 3B). Figure 3C shows that dialyzing conditioned media from LPS-treated macrophages only partially prevented the inhibition of ISC_Δamil._ (−60%; *p* = 0.001). However, the inhibition of ISC_Δamil_ was completely abolished when ATII cells were pretreated with L-NMMA. These results indicate that not only NO released from MΦ but also NO produced by rA2 causes the inhibition of rA2 Na transport by LPS-stimulated MΦ.

### 2.3. ENaC Surface Abundance

**Figure 4:**
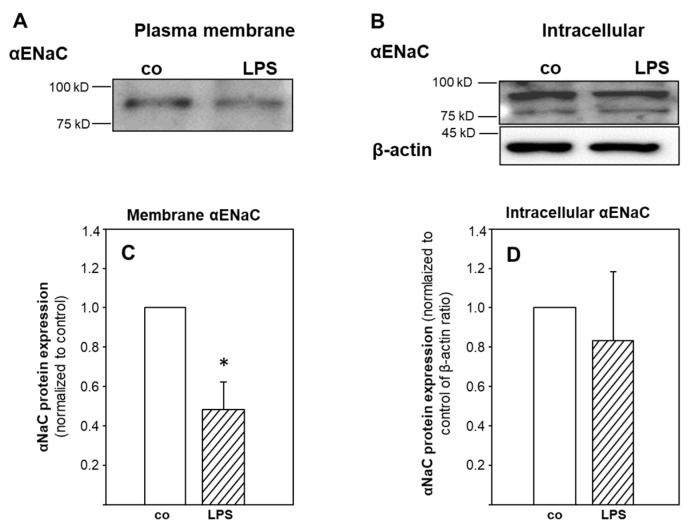
Plasma membrane and intracellular αENaC protein expression of rA2 cells treated with LPS stimulated macrophage conditioned media (Figure S6). Conditioned media from LPS stimulated macrophages were kept on primary rA2 cell monolayers for 24 h. Biotinylated proteins represent the apical membrane fraction, non-biotinylated is considered intracellular. Representative immunoblots showing αENaC (**A**,**C**) surface expression (biotinylated). (**B**,**D**) Intracellular αENaC (non-biotinylated). β-actin was not detected in the surface (biotinylated) membrane fraction but only in the intracellular pool. * effect of LPS treated macrophage conditioned media, *p* = 0.03.

To further explore possible mechanisms of ENaC-inhibition by LPS-treated macrophages, we measured the membrane abundance of αENaC protein, which is the subunit required for pore formation and Na-transport [24]. Figure 4A shows that conditioned media from LPS-stimulated macrophages decreased the membrane–surface expression of αENaC (−50%; *p* = 0.03), whereas intracellular αENaC was not affected, indicating increased internalization and degradation of αENaC.

### 2.4. Hypoxia

**Figure 5:**
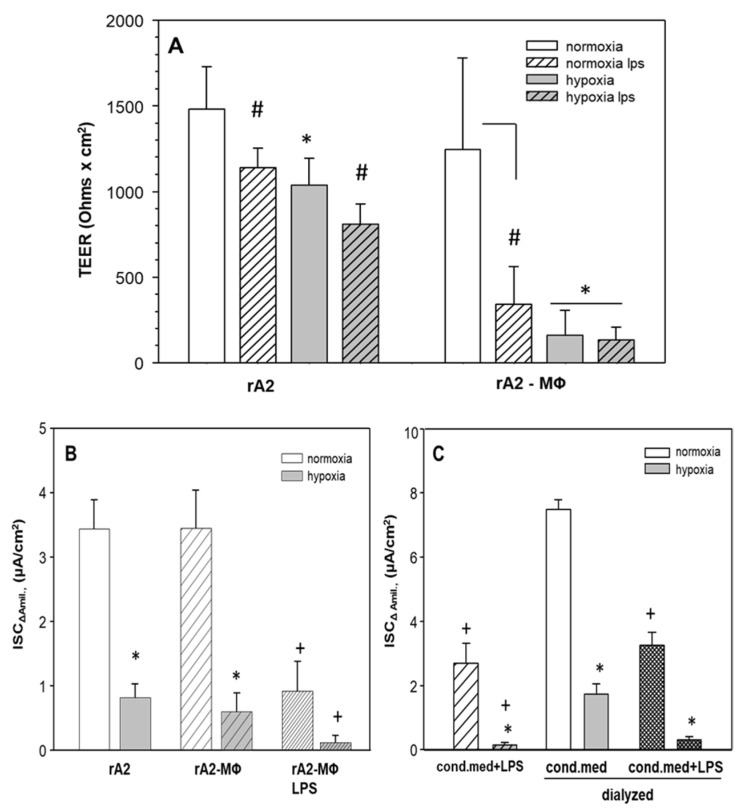
Effects of hypoxia on transepithelial resistance and ion transport of rA2 cells in mono- and co-culture with alveolar macrophages and of dialysis of conditioned medium obtained from LPS stimulated macrophages. Mono and co-cultures of rA2 cells with alveolar macrophages were prepared as described in Methods section and treated with LPS (1 μg/mL) and kept in hypoxia for 24 h. Transepithelial electrical resistance (TEER) was measured by EVOM (Ohms × cm^2^) (**A**,**B**). Dialyzed and non-dialyzed conditioned macrophage media treated with LPS (1 µg/mL) was prepared as described in Methods section and kept on rA2 monolayers for 24 h in normoxia or hypoxia (**C**). ENaC activity was measured as the amiloride (10 µM) sensitive component of I*eq* in Ussing chambers (ISC_Δamil_). Mean values ± SD of 4–8 experiments from at least 3 independent preparations of primary rA2 cells. # *p* < 0.001 effect of LPS in (A), * *p* < 0.001 effect of hypoxia, + *p* < 0.001 effect of LPS treatment compared to relevant controls. MΦ: rat alveolar macrophages; rA2: rat alveolar epithelial cells.

Because hypoxia, which often occurs in lung inflammation also inhibits alveolar reabsorption, we tested whether the effects were additive. Figure 5A shows that hypoxia decreased TEER of rA2 mono-cultures by 30% (*p* < 0.001). Hypoxia of rA2—MΦ co-cultures decreased TEER (−80%) even further. LPS-treatment decreased TEER in normoxia (*p* < 0.001) and hypoxia (*p* = 0.008) in both monolayers and co-cultures. Figure 5B shows that hypoxia decreased ENaC activity in rA2 monolayers as well as in rA2—MΦ co-cultures (−80%; *p* < 0.001) in absence of LPS. LPS treatment in hypoxia further decreased ENaC activity in the co-cultures (residual activity 5% of control; *p* = 0.002). 

We also measured ENaC activity in normoxic and hypoxic rA2 cells that were exposed to dialyzed and non-dialyzed macrophage conditioned media. Consistent with results from the co-culture experiments, hypoxia decreased ENaC activity (*p* = 0.001), and further decrease was observed, when cells were treated with LPS conditioned media (Figure 5C; *p* = 0.001). Dialyzing LPS-treated macrophage conditioned media did not prevent the massive inhibition of ENaC-activity by the combination of LPS-conditioned MΦ-media and hypoxia (*p* = 0.06), similar to what was seen in the co-cultures (Figure 5B).

## 3. Discussion

Studying the effects of inflammation on alveolar epithelial Na-reabsorption in-vitro requires the combined culturing of alveolar macrophages with alveolar epithelial cell monolayers because the co-culture system accounts for cell-to-cell interactions, which is essential for integrating cell-specific responses, and to simulate as close as possible the in-vivo situation. Here, we report results from co-culturing NR8383 rat alveolar macrophages (MΦ) with rat primary alveolar epithelial cells (rA2) in a transwell-system to examine the effects of endotoxin (LPS)-induced inflammation on alveolar epithelial Na-transport by ENaC. Our results show the typical inflammatory response of MΦ-stimulation, which is propagated to the rA2 cells, where it causes the inhibition of ENaC-activity. Inhibition most likely depends on NO produced by MΦ. In addition, NO produced from rA2 also contributes because the decreased ENaC activity was prevented by inhibiting inducible nitric oxide synthase (iNOS) in rA2 when conditioned media from LPS-stimulated MΦ were used, even after NO and its metabolites had been removed by dialysis. Furthermore, we show here that hypoxia, which is common finding in inflammatory lung diseases, aggravates inflammation-induced ENaC inhibition by mechanisms independent of NO.

It has been shown previously in mono-cultures of rA2 that inflammatory mediators inhibit ENaC and alveolar fluid clearance by MAP kinase dependent mechanisms [25]. LPS-treatment of rA2 inhibited ENaC by extracellular ATP and P2Y2-dependent signaling [26]. There are also reports that rA2 produces inflammatory cytokines upon stimulation with LPS [6,27]. Although these experiments were all performed on alveolar epithelial cell monolayers without the direct communication between macrophages and alveolar epithelium, results indicate that, upon stimulation with LPS, inflammatory cytokines released from MΦ and/or rA2 seem to inhibit ENaC. The mechanisms are less clear. The typical inflammatory response of MΦ includes MAP kinase-dependent stimulation of the expression of various cytokines and of iNOS resulting in the production of NO [22,28,29,30]. Furthermore, treatment of oocytes over-expressing ENaC with NO-donors inhibits ENaC-activity, likely by formation of the pro-oxidant peroxynitrite [31]. This indicates that externally produced NO adversely affects Na-transport. While the mechanisms are unclear; nitration might play a role, similar to what has been shown for NO-dependent Na/K-ATPase inhibition [32] and adenoviral infection in the lung [33]. Although these results indicate a series of events involved in the inflammation induced inhibition of alveolar epithelial Na-transport, it remains unclear which of the signals are essential, and in which of the cellular compartments they are produced.

To address this issue, we simulated the in-vivo condition by using co-cultures of MΦ and rA2 in a transwell-system. This model allowed us to study the sidedness of the effect (MΦ apical or basolateral) and to interfere with processes occurring within the MΦ and the rA2 to obtain information of individual cellular responses and crosstalk, while, at the same time, transepithelial transport Na-transport could be measured in Ussing chambers.

### 3.1. Inflammatory Signaling and ENaC-Activity

Previous studies demonstrated that pro- and anti-inflammatory molecules, chemokines [22,28], reactive oxygen species (ROS) and nitrate release from activated alveolar macrophages [29,30], upon bacterial or viral infections [28], impair lung barrier function [34,35], decrease the rate of alveolar fluid reabsorption [25,36,37] and cause pulmonary edema. Our results on MΦ also show that stimulation with LPS increased mRNA-expression of TNF-α, IL-6 and iNOS (Figure 2; Supplementary Figure S2A,B) and increased the release of NO (Figure 2C and Figure S4). Increased cytokine mRNA-expression in MΦ could be prevented with MAPK and ERK1/2 inhibitors (not shown) and the release of NO from MΦ was prevented by inhibiting iNOS activity with L-NMMA, indicating that MΦ shows the typical pro-inflammatory response [22].

LPS-treatment of MΦ significantly reduced alveolar epithelial Na-transport (ISC_Δamil_) in MΦ/rA2 co-cultures (Figure 1B). We also tested whether conditioned media from LPS treated MΦ cause ENaC inhibition as in co-cultures. Figure 1C shows that these conditioned media also inhibited ENaC activity by ~50%, which appears less pronounced than in co-culture experiments. This might indicate that cell–cell communication and not-yet-identified signaling is required to observe full inhibition, and that soluble molecules propagate inflammatory signaling to rA2. MΦ-derived cyto-/chemokines might cause the inhibition of ENaC, as the inhibition of alveolar Na-transport by TNF-α, TGF-β, and IL-1β has been described [25,38,39,40].

Because treatment of MΦ/rA2-co-cultures with L-NMMA and the removal of NO and its derivatives from MΦ-conditioned media by dialysis prevented most of the inhibition of Na-transport across rA2 (Figure 2C and Figure 3), it is likely that NO released from MΦ caused Na-transport inhibition. This notion is supported by results showing inhibition of alveolar Na-transport by short- and long-acting NO-donors [29,41]; we confirm these findings in our experimental setup (Supplementary Figure S5). However, even after removing NO and its metabolites by dialysis, conditioned media from LPS-stimulated MΦ still cause a significant reduction of ISC_Δamil_ across rA2-monolayers after 24-h treatment (Figure 3C). This effect was prevented by inhibiting iNOS in rA2-monolayers, indicating that, in addition to NO from MΦ, rA2 also produces sufficient NO to adversely affect Na-transport. We cannot distinguish whether the increase in NO is caused by a direct effect of residual LPS or by signaling via MΦ-derived cyto-/chemokines present in the conditioned media after dialysis. Resolving this question might be of significance because of apparently contradictory results. Boncoeur and colleagues have shown that 4-h incubation of rA2 with LPS increased NO-production by PKC-dependent mechanisms [42]. However, in our experimental system, an increase in NO-production was not seen earlier than after 12 h of LPS-treatment by both rA2 and MΦ (Supplementary Figure S4). Figure 1B also shows that 24-h LPS treatment of rA2 cells did not inhibit ENaC activity, which contrasts with the findings of Boncoeur and colleagues, who reported decreased activity of ENaC already after 4 h treatment of rA2 cells with LPS from *P*. *aeruginosa* [42]. Differences in the culturing conditions, source of LPS, and the time of exposure might explain this discrepancy.

### 3.2. Mechanisms of Na-Transport Inhibition

Although the role of NO derived from MΦ and rA2 appears to be the dominant candidate for explaining the inhibition of rA2 Na-transport, other mechanisms might contribute or even mediate or enhance the effects of NO. Peroxynitrite, which is a derivative of NO formed after reaction with ROS, has been shown to inhibit Na-transport in rA2 [29,43]. However, although TEMPO and urate decreased basal ion transport activity, particularly in rA2 cell mono-cultures, neither of the two substances affected transport inhibition by LPS in the rA2 cells co-cultured with MΦ (data not shown). We did not test the effects of ROS-scavengers on rA2, when these cells were treated with dialyzed media from LPS-stimulated MΦ.

A decreased membrane abundance of ENaC might explain decreased transport activity; however, the results are controversial. We show here that 24-h treatment with conditioned media from LPS-treated MΦ decreased the surface abundance of αENaC but not the intracellular amount (Figure 4). This might indicate that mediators released from macrophages caused internalization. Our result is in line with findings by Roux and colleagues [44], who showed that 4-h treatment of rA2 cells with IL-1β decreased αENaC membrane expression and activity, which was reversed by inhibiting p38 MAPK signaling. In contrast, Boncoeur and colleagues [26] reported decreased ENaC activity without loss of ENaC membrane abundance by direct and short-term application of LPS from *P*. *aeruginosa* to rA2 cells. It is thus not clear what causes decreased activity, internalization, breakdown of internalized ENaC, or both. Again, treatment conditions might explain the discrepancies. Althaus and colleagues [32] showed that NO nitrosylates the Na/K-ATPase in H441 airway epithelial cells, which is paralleled by decreased Na/K-ATPase activity, that NO-dependent processes impair vectorial Na-transport across lung epithelia. Preventing the NO-reaction with thiol-groups also prevented Na/K-ATPase inhibition, which points to a causal relation [32]. It is not clear whether a similar mechanism affects ENaC.

Another point of discussion might be the role of TNF-α. It has been shown that TNF-α causes the inhibition of ENaC by MAP kinase-dependent pathways [25]; but there are discrepant results. Elia and colleagues [45] have shown that a lectin-like domain of the TNF-α molecule can directly interact with ENaC, which may stimulate edema clearance in mild-to-moderate lung injury by increased ENaC mediated transport [46]. It is unclear which condition favors the one or the other response. It appears that in severe lung injury other mediators such as NO, proteases, and oxidants involved in different signaling pathways may also participate along with alveolar epithelial cell injury [7]. Recent reports showing the resolution of lung edema and injury in-vivo and in-vitro through ALX/cAMP/PI3K/Nedd4-2 and WNK4/SPAK axis [47,48] support this argument. For example, Resolvin conjugates in tissue regeneration 1 (RCTR1), a series of docosahexaenoic acid–derived lipid mediators which can be released from macrophages when externally applied to LPS induced lung injury model upregulates ENaC abundance and improves alveolar fluid clearance [47]. Mesenchymal stem cells secrete a wide range of cytokines, growth factors, and microRNAs [49] attenuating edematous lung injury via enhancing γ-ENaC expression, through activating PI3K/AKT signaling [36]. Therefore, depending on the severity of the lung injury, the cause, and maybe the time elapsed from the onset of the disease, different signaling pathways might be involved in the regulation of alveolar fluid clearance.

### 3.3. Hypoxia and Inflammation

ARDS not only inhibits alveolar epithelial Na- and water reabsorption, but it also causes alveolar and interstitial edema, which impair the diffusion of oxygen to and across the alveolar epi- and endothelium causing hypoxia. It is thus of importance to understand how inflammation and hypoxia interact in their ways to modulate alveolar reabsorption. We and others previously have reported that hypoxia inhibits ion transport in rA2 cells both in-vivo and in -vitro on cultured rA2 monolayers [8,15,17,18,50,51]. Hypoxic transport inhibition is achieved by internalization of ENaC [50,51] and Na/K-ATPase [52]. In order to examine the combined effects of hypoxia and LPS in our model, co-cultures of MΦ and rA2 and rA2-mono-cultures were treated with LPS or conditioned media from LPS-stimulated MΦ and were then exposed to hypoxia for 24 h. Hypoxia and LPS treatment of rA2 monolayers slightly decreased TEER, whereas LPS treatment of co-cultures further decreased TEER in hypoxia (Figure 5A). Here we report for the first time that the effects of hypoxia and inflammation nearly completely abolished Na-transport across rA2, both in the co-culture model and when rA2 monolayers were treated with conditioned media, dialyzed or not (Figure 5B,C). On the other hand, conditioned media from MΦ treated with LPS in hypoxia did not impair Na-transport, when rA2 were kept in normoxia (Supplementary Figure S1) indicating the dominant inhibitory role of hypoxia on alveolar epithelial Na-reabsorption.

### 3.4. Conclusions

**Figure 6:**
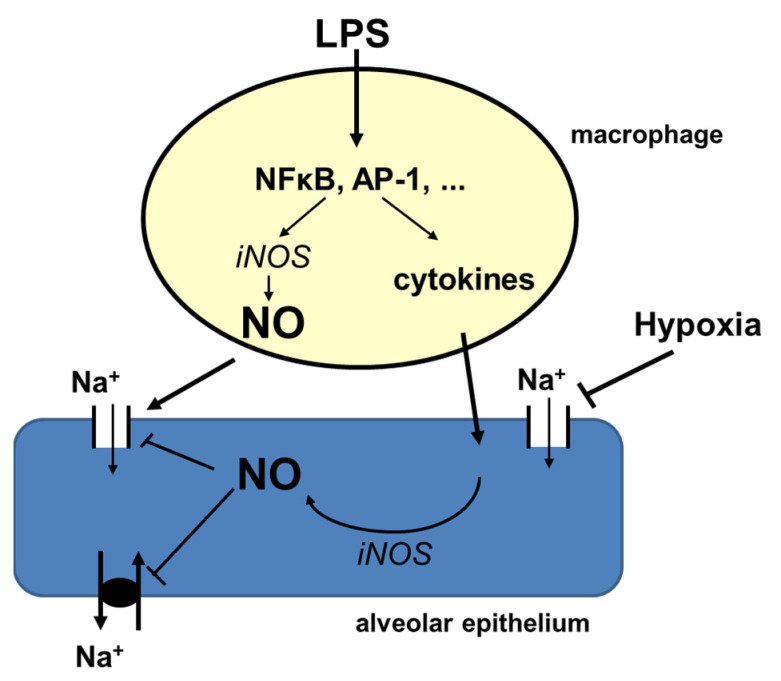
Alveolar macrophages release cytokines, chemokines, nitrite and related products upon stimulation with LPS which impair alveolar epithelial Na-transport by NO-dependent mechanisms that can be prevented by blocking iNOS activity in macrophages. iNOS is also activated in alveolar epithelial cells in response to cytokine and nitrite related products released from activated macrophages. Hypoxia by its direct effects on the alveolar epithelium additively inhibits alveolar epithelial barrier tightness and ion transport in lung inflammation.

Taken together our results indicate that the LPS-stimulation of alveolar macrophages impairs alveolar epithelial Na-transport by NO-dependent mechanisms, where the majority of NO comes from MΦ, and a smaller amount from rA2 upon signaling from the MΦ (Figure 6). Hypoxia aggravates impairment of reabsorption in this state of inflammation apparently independent of inflammation-induced signaling (Figure 6). It is of great clinical significance to understand this interaction between both alveolar cell types in ARDS to improve impaired ENaC activity, which is essential for the outcome of the disease. Therefore, targeting iNOS activity in alveolar cells in the inflamed lung may bring a new approach to improve impaired Na-transport. Future studies are needed to test this hypothesis.

As an additive treatment NO is often considered to have a favorable effect on the distribution of lung blood flow in the hypoxic lung, which might improve systemic hypoxia [53]. In addition to vasodilatory effect, its antiviral activity against SARS-CoV-2 infection has been also suggested [54]. However, due to potentially adverse effects of NO-related products, particularly of peroxynitrite, NO treatment strategies of patients with respiratory distress and hypoxemia may have the high risk of exacerbating inflammation [55,56,57], amongst others, by impairing epithelial reabsorption [32].

## 4. Materials and Methods

### 4.1. Preparation of Rat Lung Primary Alveolar Epithelial Cells (rA2)

Primary rA2 cells were isolated from lungs of normoxic male rats (Sprague–Dawley, 170–200 g) as described earlier [8,58]. Experiments were approved by the Animal Protection Committee of the University of Heidelberg and by the Regierungspräsidium Karlsruhe, Germany (T-21/09; T-64/15). Briefly, rats were anesthetized (100 mg/kg Na-pentobarbital, i.e., Trapanal, Byk Gulden, Konstanz, Germany) and lungs were perfused with a buffer composed of (in mM) 136 NaCl, 5.3 KCl, 5.6 glucose, 2.6 NaH_2_PO_4,_ 10 HEPES, 0.15 EGTA, pH 7.4 at RT, while being ventilated. rA2 cells were isolated by elastase digestion (Elastin Products, Owensville, MO, USA), mincing of lung tissue, differential filtration, and differential adhesion in IgG-coated plates. Non-adherent cells were suspended in DMEM-N, i.e., DMEM supplemented with 10% neonatal calf serum, glutamine (4 mM) and gentamycin (50 µg/mL) and were plated on the side of the nucleopore filters facing the inner compartment at a density of 1.5 × 10^6^ cells/cm². Both purity and viability of rA2 cells were >90%. Cells were cultured in normoxia (room air with 5% CO_2_) in a liquid–liquid interface, which allows the collection of culture medium for the analysis of released signaling molecules.

### 4.2. NR8383 Rat Alveolar Macrophage Cell Line (NR8383)

Because primary rat lung macrophages collected by bronco-alveolar lavage appeared to be in a stimulated state, experiments presented here were performed with the rat lung derived NR8383 alveolar macrophage cell line (ATCC, Manassas, VA, USA). Cells were grown in 75 cm^2^ tissue culture flasks using DMEM-N (see above, rA2 cells). Both adherent and floating cells were harvested and mixed for further use between passages 6 and 12.

### 4.3. Co-Culture of rA2 with NR8383

Two days after seeding, rA2 cells had reached confluence indicated by a transepithelial electrical resistance (TEER) >250 Ohms × cm^2^. In preliminary experiments, NR8383 macrophages were added to the apical or basolateral side of the transwells and were co-cultured for 24 h before other treatments. Since results on ion transport and gene expression were similar to the apical and basolateral placement of NR8383, and since no clean separation of cell types for RNA preparation was possible in the mixed culture, all further experiments were performed with the macrophages (3 × 10^5^ cells in 1.5 mL) added to the basolateral compartment.

### 4.4. Preparation of Macrophage-Conditioned Media and Dialysis

NR8383 grown in 75 cm^2^ flasks for two days were treated (e.g., LPS, 1 µg/mL; L-NMMA, 1 mM, controls with respective solvent) for 24 h. Culture media were collected, non-adherent cells were removed by centrifugation, and media were stored frozen at −80 °C until use with rA2 cell mono-cultures. To study possible effects of NO, nitrite and related products released from LPS stimulated macrophages, conditioned media from control and LPS-stimulated NR8383 was dialyzed using MW-500 Dalton cut-off dialysis tubing (Spectrum, Roth, Germany) against DMEM overnight and against fresh DMEM for another 4 h. Dialyzed medium was sterile filtered and kept frozen at −80 °C until use.

### 4.5. Treatments

rA2 cells reached confluence on day two after cell preparation. For co-cultures, NR8383 were kept on the basolateral side of the rA2 cells for 24 h. The next day, NR8383 were treated with LPS (1 µg/mL), L-NMMA (1 mM) (both from Sigma-Aldrich, Darmstadt, Germany), and exposed to hypoxia (1.5% O_2_; 5% CO_2_, rest N_2_) using an O_2_/CO_2_-controlled incubator (Binder, Tuttlingen, Germany) for up to 48 h as indicated. Control cultures remained in normoxia. Experiments were also performed on rA2 mono-cultures. In some experiments, normoxic rA2 cell monolayers were treated with conditioned media from LPS-treated macrophage in normoxia and hypoxia (1.5% O_2_; 5% CO_2_, rest N_2_).

### 4.6. Electrophysiological Measurements

The tightness of the mono- and co-cultures was measured as the transepithelial electrical resistance (TEER) using an epithelial volt-ohm-meter and chopstick electrodes (EVOM; World Precision Instruments, Sarasota, FL, USA).

Ion transport activity was measured in mono-and co-cultures after mounting transwells into Ussing chambers as described earlier [8]. Filters were bathed with a pre-warmed Krebs buffer composed of (in mM) 141 NaCl, 5.4 KCl, 0.78 NaH_2_PO_4_, 1.8 CaCl_2_, 0.8 MgCl_2_, 5 glucose and 15 HEPES, pH 7.4 at 37 °C). After equilibration (10 min, 37 °C; open circuit conditions), the transepithelial potential was clamped to 0 mV and the short circuit current (ISC_tot_) was recorded with an automated voltage clamp unit (W.Nagel, Munich, Germany). The component of ISC inhibited by amiloride (10 µM; ISC_Δamil_) served as an indicator of transport mediated by epithelial Na-channels (ENaC). The capacity of the Na/K-ATPase was the ISC after permeabilization of the apical membrane with amphotericin B (5 µM; ISC_ampho_).

### 4.7. RNA Isolation and Quantitative RT-PCR

rA2 cells were washed once with phosphate buffered saline (PBS), macrophages were scraped off the surface, packed by centrifugation and washed with PBS. Cells were lyzed with RLT reagent (Qiagen, Hilden, Germany), and total RNA was isolated using the RNeasy micro kit (Qiagen, Hilden, Germany) according to the manufacturer’s instructions. RNA (0.1 µg) was transcribed with Superscript II reverse transcriptase (Invitrogen, Life Technologies, Langenselbold, Germany) using random hexamere primers (Roche, Mannheim, Germany). Real time quantitative PCR was performed in the LightCycler^®^ (Model 480, Roche, Mannheim, Germany). The ABsolute QPCR SYBRgreen mix (Thermo Scientific, Schwerte, Germany) was used with QuantiTect^®^-primers (QuantiTect^®^, Qiagen, Hilden, Germany) for the detection of iNOS and cytokines; 28S-rRNA was used as a housekeeping gene. The 2^−^^△△Ct^ method was used to analyze the relative mRNA abundance [59].

### 4.8. Cytokine Measurements

Levels of TNF-α and IL-6 in control and LPS stimulated macrophage conditioned medium (dialyzed and non-dialyzed) were quantified by the Luminex multiplex bead assay (Bio-Rad, Munich, Germany). Measurements were performed on a BioPlex200 System using the Bio-Plex Pro Cytokine Reagent Kit and Bio-Plex Pro Rat Cytokine sets (both Bio-Rad) according to manufacturer’s instructions. Cytokine levels are given as Fluorescence intensity (FI), which was acquired using Bio-Plex Manager software version 6.1 (Bio-Rad).

### 4.9. Cell Surface Biotinylation and Western Blotting

The surface expression of αENaC from LPS stimulated macrophage conditioned media was measured after biotinylation of apical membrane proteins [60,61]. Briefly, cells were washed with ice-cold PBS, incubated with NHS-SS-biotin (Pierce, Schwerte, Germany) for 20 min at 4 °C, and washed with PBS containing glycine (100 mM). Cells were lyzed with lysis buffer (1% Triton X-100, 150 mM NaCl, 5 mM EDTA, 50 mM Tris, pH 7.5), and lysates were centrifuged (2 min, 13,000 rpm, 4 °C). Supernatants containing 200 µg of total protein were incubated with Streptavidin-agarose beads (Pierce, Schwerte, Germany) overnight at 4 °C. Beads were pelleted by centrifugation. Supernatants (i.e., the non-biotinylated fraction) containing the intracellular proteins were collected and stored frozen. Biotinylated proteins representing the apical membrane fraction were eluted from the beads by heating at 95 °C, 5 min, in a 4x-sample buffer. Non-biotinylated and biotinylated samples were used for Western blotting. Proteins were separated on 10% SDS-PAGE and transferred onto PVDF membranes for Western blot analysis using the α-ENaC antibody (rabbit, PA1-920) from Thermo Scientific (Schwerte, Germany) with β-actin as a reference. Secondary antibodies, conjugated with horseradish peroxidase and enhanced chemiluminescence (GE Healthcare/Amersham, Freiburg, Germany), were used for detection. Band densities were measured using the Image J software (NIH, Bethesda, MD, USA).

### 4.10. Other Measurements

Nitrite was determined to be an indicator of the NO formation of macrophages and rA2 cells using a modified Griess reagent (Sigma-Aldrich, Darmstadt, Germany). The absorbance was measured with a spectrophotometer at 555 nm; the concentration was calculated using sodium nitrite as standard.

### 4.11. Statistical Evaluation

Results are shown as mean ± SE or SD as indicated. Statistical analysis was performed by analysis of variance (ANOVA) for repeated measures and pair-wise multiple comparisons (LSD) or *t*-tests as indicated using the SigmaPlot (Systat Inc., Erkrath, Germany) software package. The level of statistical significance was *p* < 0.05.

## Supplementary Materials

The following supporting information can be downloaded at: https://www.mdpi.com/article/10.3390/ijms23158315/s1.

## References

[1] Delclaux C., Azoulay E. (2003). Inflammatory response to infectious pulmonary injury. Eur. Respir. J..

[2] Bode J.G., Ehlting C., Haussinger D. (2012). The macrophage response towards LPS and its control through the p38(MAPK)-STAT3 axis. Cell. Signal..

[3] Chen Z.T., Li S.L., Cai E.Q., Wu W.L., Jin J.S., Zhu B. (2003). LPS induces pulmonary intravascular macrophages producing inflammatory mediators via activating NF-kappaB. J. Cell. Biochem..

[4] Wang W., Ye L., Ye L., Li B., Gao B., Zeng Y., Kong L., Fang X., Zheng H., Wu Z. (2007). Up-regulation of IL-6 and TNF-alpha induced by SARS-coronavirus spike protein in murine macrophages via NF-kappaB pathway. Virus Res..

[5] Haddad I.Y., Zhu S., Crow J., Barefield E., Gadilhe T., Matalon S. (1996). Inhibition of alveolar type II cell ATP and surfactant synthesis by nitric oxide. Am. J. Physiol..

[6] Trapnell B.C., Whitsett J.A. (2002). Gm-CSF regulates pulmonary surfactant homeostasis and alveolar macrophage-mediated innate host defense. Annu. Rev. Physiol..

[7] Hamacher J., Hadizamani Y., Borgmann M., Mohaupt M., Mannel D.N., Moehrlen U., Lucas R., Stammberger U. (2017). Cytokine-Ion Channel Interactions in Pulmonary Inflammation. Front. Immunol..

[8] Mairbäurl H., Mayer K., Kim K.J., Borok Z., Bärtsch P., Crandall E.D. (2002). Hypoxia decreases active Na transport across primary rat alveolar epithelial cell monolayers. Am. J. Physiol..

[9] Mairbäurl H. (2006). Role of alveolar epithelial sodium transport in high altitude pulmonary edema (HAPE). Respir. Physiol. Neurobiol..

[10] Clerici C. (1998). Sodium transport in alveolar epithelial cells: Modulation by O2 tension. Kidney Int..

[11] Ogawa S., Koga S., Kuwabara K., Brett J., Morrow B., Morris S.A., Bilezikian J.P., Silverstein S.C., Stern D. (1992). Hypoxia-induced increased permeability of endothelial monolayers occurs through lowering of cellular cAMP levels. Am. J. Physiol..

[12] Dehler M., Zessin E., Bärtsch P., Mairbäurl H. (2006). Hypoxia causes permeability edema in the constant-pressure perfused rat lung. Eur. Respir. J..

[13] Chao J., Wood J.G., Gonzalez N.C. (2009). Alveolar hypoxia, alveolar macrophages, and systemic inflammation. Respir. Res..

[14] Matthay M.A., Clerici C., Saumon G. (2002). Active fluid clearance from distal airspaces. J. Appl. Physiol..

[15] Guney S., Schuler A., Ott A., Höschele S., Baloglu E., Bärtsch P., Mairbäurl H. (2007). Dexamethasone prevents transport inhibition by hypoxia in rat lung and alveolar epithelial cells by stimulating activity and expression of Na+/K+-ATPase and epithelial Na+ channels. Am. J. Physiol..

[16] Baloglu E., Reingruber T., Bärtsch P., Mairbäurl H. (2011). beta2-Adrenergics in hypoxia desensitize receptors but blunt inhibition of reabsorption in rat lungs. Am. J. Respir. Cell Mol. Biol..

[17] Planes C., Escoubet B., BlotChabaud M., Friedlander G., Farman N., Clerici C. (1997). Hypoxia downregulates expression and activity of epithelial sodium channels in rat alveolar epithelial cells. Am. J. Respir. Cell Mol. Biol..

[18] Vivona M.L., Matthay M.A., Chabaud M.B., Friedlander G., Clerici C. (2001). Hypoxia reduces alveolar epithelial sodium and fluid transport in rats: Reversal by beta-adrenergic agonist treatment. Am. J. Respir. Cell Mol. Biol..

[19] Price L.C., McAuley D.F., Marino P.S., Finney S.J., Griffiths M.J., Wort S.J. (2012). Pathophysiology of pulmonary hypertension in acute lung injury. Am. J. Physiol. Lung Cell. Mol. Physiol..

[20] Ware L.B., Matthay M.A. (2001). Alveolar fluid clearance is impaired in the majority of patients with acute lung injury and the acute respiratory distress syndrome. Am. J. Respir. Crit. Care Med..

[21] Matthay M.A. (2002). Alveolar fluid clearance in patients with ARDS: Does it make a difference?. Chest.

[22] Thorley A.J., Ford P.A., Giembycz M.A., Goldstraw P., Young A., Tetley T.D. (2007). Differential regulation of cytokine release and leukocyte migration by lipopolysaccharide-stimulated primary human lung alveolar type II epithelial cells and macrophages. J. Immunol..

[23] Whitsett J.A., Alenghat T. (2015). Respiratory epithelial cells orchestrate pulmonary innate immunity. Nat. Immunol..

[24] Shehata M.F. (2010). The Epithelial Sodium Channel alpha subunit (alpha ENaC) alternatively spliced form “b” in Dahl rats: What’s next?. Int. Arch. Med..

[25] Eaton D.C., Helms M.N., Koval M., Bao H.F., Jain L. (2009). The contribution of epithelial sodium channels to alveolar function in health and disease. Annu. Rev. Physiol..

[26] Boncoeur E., Tardif V., Tessier M.C., Morneau F., Lavoie J., Gendreau-Berthiaume E., Grygorczyk R., Dagenais A., Berthiaume Y. (2010). Modulation of epithelial sodium channel activity by lipopolysaccharide in alveolar type II cells: Involvement of purinergic signaling. Am. J. Physiol..

[27] Haddad J.J., Land S.C. (2002). Redox signaling-mediated regulation of lipopolysaccharide-induced proinflammatory cytokine biosynthesis in alveolar epithelial cells. Antioxid. Redox Signal..

[28] Ward P.A. (1997). Phagocytes and the lung. Ann. N. Y. Acad. Sci..

[29] Guo Y., Duvall M.D., Crow J.P., Matalon S. (1998). Nitric oxide inhibits Na + absorption across alveolar type II monolayers. Am. J. Physiol..

[30] Jain L., Chen X.J., Brown L.A., Eaton D.C. (1998). Nitric oxide inhibits lung sodium transport through a cGMP-mediated inhibition of epithelial cation channels. Am. J. Physiol..

[31] Helms M.N., Yu L., Malik B., Kleinhenz D.J., Hart C.M., Eaton D.C. (2005). Role of SGK1 in nitric oxide inhibition of ENaC in Na+-transporting epithelia. Am. J. Physiol. Cell Physiol..

[32] Althaus M., Pichl A., Clauss W.G., Seeger W., Fronius M., Morty R.E. (2011). Nitric oxide inhibits highly selective sodium channels and the Na+/K+-ATPase in H441 cells. Am. J. Respir. Cell Mol. Biol..

[33] Zsengeller Z.K., Ross G.F., Trapnell B.C., Szabo C., Whitsett J.A. (2001). Adenovirus infection increases iNOS and peroxynitrite production in the lung. Am. J. Physiol. Lung Cell. Mol. Physiol..

[34] Farley K.S., Wang L.F., Law C., Mehta S. (2008). Alveolar macrophage inducible nitric oxide synthase-dependent pulmonary microvascular endothelial cell septic barrier dysfunction. Microvasc. Res..

[35] Farley K.S., Wang L., Mehta S. (2009). Septic pulmonary microvascular endothelial cell injury: Role of alveolar macrophage NADPH oxidase. Am. J. Physiol. Lung Cell. Mol. Physiol..

[36] Hua Y., Han A., Yu T., Hou Y., Ding Y., Nie H. (2022). Small Extracellular Vesicles Containing miR-34c Derived from Bone Marrow Mesenchymal Stem Cells Regulates Epithelial Sodium Channel via Targeting MARCKS. Int. J. Mol. Sci..

[37] Iles K.E., Song W., Miller D.W., Dickinson D.A., Matalon S. (2009). Reactive species and pulmonary edema. Expert Rev. Respir. Med..

[38] Willis B.C., Kim K.J., Li X., Liebler J., Crandall E.D., Borok Z. (2003). Modulation of ion conductance and active transport by TGF- beta 1 in alveolar epithelial cell monolayers. Am. J. Physiol..

[39] Dagenais A., Frechette R., Yamagata K., Yamagata T., Carmel J.F., Clermont M.E., Brochiero E., Masse C., Berthiaume Y. (2004). Downregulation of ENaC activity and expression by TNF-à in alveolar epithelial cells. Am. J. Physiol..

[40] Frank J., Roux J., Kawakatsu H., Su G., Dagenais A., Berthiaume Y., Howard M., Canessa C.M., Fang X., Sheppard D. (2003). Transforming growth factor-beta1 decreases expression of the epithelial sodium channel alphaENaC and alveolar epithelial vectorial sodium and fluid transport via an ERK1/2-dependent mechanism. J. Biol. Chem..

[41] Nielsen V.G., Baird M.S., Chen L., Matalon S. (2000). DETANONOate, a nitric oxide donor, decreases amiloride- sensitive alveolar fluid clearance in rabbits. Am. J. Respir. Crit. Care Med..

[42] Boncoeur E., Bouvet G.F., Migneault F., Tardif V., Ferraro P., Radzioch D., de Sanctis J.B., Eidelman D., Govindaraju K., Dagenais A. (2013). Induction of nitric oxide synthase expression by lipopolysaccharide is mediated by calcium-dependent PKCalpha-beta1 in alveolar epithelial cells. Am. J. Physiol. Lung Cell. Mol. Physiol..

[43] Matalon S., Hu P., Ischiropoulos H., Beckman J.S. (1994). Peroxynitrite Inhibition of Oxygen Consumption and Ion Transport in Alveolar Type II Pneumocytes. Chest.

[44] Roux J., Kawakatsu H., Gartland B., Pespeni M., Sheppard D., Matthay M.A., Canessa C.M., Pittet J.F. (2005). Interleukin-1 beta decreases expression of the epithelial sodium channel alpha-subunit in alveolar epithelial cells via a p3 8 MAPK-dependent signaling pathway. J. Biol. Chem..

[45] Elia N., Tapponnier M., Matthay M.A., Hamacher J., Pache J.C., Brundler M.A., Totsch M., De Baetselier P., Fransen L., Fukuda N. (2003). Functional identification of the alveolar edema reabsorption activity of murine tumor necrosis factor-alpha. Am. J. Respir. Crit. Care Med..

[46] Yang G., Hamacher J., Gorshkov B., White R., Sridhar S., Verin A., Chakraborty T., Lucas R. (2010). The Dual Role of TNF in Pulmonary Edema. J. Cardiovasc. Dis. Res..

[47] Yang Q., Xu H.R., Xiang S.Y., Zhang C., Ye Y., Shen C.X., Mei H.X., Zhang P.H., Ma H.Y., Zheng S.X. (2021). Resolvin Conjugates in Tissue Regeneration 1 Promote Alveolar Fluid Clearance by Activating Alveolar Epithelial Sodium Channels and Na, K-ATPase in Lipopolysaccharide-Induced Acute Lung Injury. J. Pharmacol. Exp. Ther..

[48] Deng W., Qi D., Tang X.M., Deng X.Y., He J., Wang D.X. (2022). The WNK4/SPAK Pathway Stimulates Alveolar Fluid Clearance By Up-Regulation of Epithelial Sodium Channel In Mice with Lipopolysaccharide-Induced Acute Respiratory Distress Syndrome. Shock.

[49] Zhou Z., Hua Y., Ding Y., Hou Y., Yu T., Cui Y., Nie H. (2021). Conditioned Medium of Bone Marrow Mesenchymal Stem Cells Involved in Acute Lung Injury by Regulating Epithelial Sodium Channels via miR-34c. Front. Bioeng. Biotechnol..

[50] Baloglu E., Nonnenmacher G., Seleninova A., Berg L., Velineni K., Ermis-Kaya E., Mairbäurl H. (2020). The role of hypoxia-induced modulation of alveolar epithelial Na(+)- transport in hypoxemia at high altitude. Pulm. Circ..

[51] Planes C., BlotChabaud M., Matthay M.A., Couette S., Uchida T., Clerici C. (2002). Hypoxia and beta(2)-agonists regulate cell surface expression of the epithelial sodium channel in native alveolar epithelial cells. J. Biol. Chem..

[52] Dada L., Bertorello A., Pedemonte C., Chandel N., Sznajder J.I. (2001). Hypoxia inhibits Na,K-ATPase function by endocytosis of its à1 subunit in alveolar epithelial cells. Am. J. Respir. Crit. Care Med..

[53] Scherrer U., Vollenweider L., Delabays A., Savcic M., Eichenberger U., Kleger G.R., Fikrle A., Ballmer P.E., Nicod P., Bärtsch P. (1996). Inhaled nitric oxide for high-altitude pulmonary edema. N. Engl. J. Med..

[54] Ignarro L.J. (2020). Inhaled NO and COVID-19. Br. J. Pharmacol..

[55] Jorens P.G., Vermeire P.A., Herman A.G. (1993). L-arginine-dependent nitric oxide synthase: A new metabolic pathway in the lung and airways. Eur. Respir. J..

[56] Haddad I.Y., Pataki G., Hu P., Galliani C., Beckman J.S., Matalon S. (1994). Quantitation of nitrotyrosine levels in lung sections of patients and animals with acute lung injury. J. Clin. Investig..

[57] Sittipunt C., Steinberg K.P., Ruzinski J.T., Myles C., Zhu S., Goodman R.B., Hudson L.D., Matalon S., Martin T.R. (2001). Nitric oxide and nitrotyrosine in the lungs of patients with acute respiratory distress syndrome. Am. J. Respir. Crit. Care Med..

[58] Dobbs L.G. (1990). Isolation and culture of alveolar type II cells. Am. J. Physiol..

[59] Rao X., Huang X., Zhou Z., Lin X. (2013). An improvement of the 2^−ΔΔCT^) method for quantitative real-time polymerase chain reaction data analysis. Biostat. Bioinforma. Biomath..

[60] Gottardi C.J., Dunbar L.A., Caplan M.J. (1995). Biotinylation and assessment of membrane polarity: Caveats and methodological concerns. Am. J. Physiol..

[61] Peters D.M., Vadasz I., Wujak L., Wygrecka M., Olschewski A., Becker C., Herold S., Papp R., Mayer K., Rummel S. (2014). TGF-beta directs trafficking of the epithelial sodium channel ENaC which has implications for ion and fluid transport in acute lung injury. Proc. Natl. Acad. Sci. USA.

