# Supplementary material for: Hypoxia Aggravates Inhibition of Alveolar Epithelial Na-Transport by Lipopolysaccharide-Stimulation of Alveolar Macrophages
Source: Int J Mol Sci. 2022 Jul 27;23(15):8315. doi: 10.3390/ijms23158315 (PMC9368968; doi:10.3390/ijms23158315)
Supplement: Supplementary file 1 [file ijms-23-08315-s001.zip › ijms-1819065-supplementary.pdf]

Supplement:

Hypoxia aggravates inhibition of alveolar epithelial Na-transport by lipopolysaccharide-stimulation of alveolar macrophages

Emel Baloğlu[1,4], Kalpana Velineni[4], Ezgi Ermis-Kaya[4], Heimo Mairbäurl[2,3,4*]

1) Department of Pharmacology, Acibadem Mehmet Ali Aydinlar University School of Medicine, Istanbul, Turkey. emel.baloglu@acibadem.edu.tr

2) Medical Clinic VII, Sports Medicine, University Hospital Heidelberg, Germany

3) Translational Pneumology, University Hospital Heidelberg, Heidelberg, Germany

4) Translational Lung Research Center Heidelberg (TLRC-H), part of the German Center for Lung Research (DZL).

emel.baloglu@acibadem.edu.tr; kalpu8757@gmail.com; ezgiermis@gmail.com; heimo.mairbaeurl@med.uni-heidelberg.de

* Correspondence: heimo.mairbaeurl@med.uni-heidelberg.de; Tel.: +49 6221 56 39329

* Correspondence: heimo.mairbaeurl@med.uni-heidelberg.de; tel.: +49 6221 56 39329

Supplementary Figure S1A:

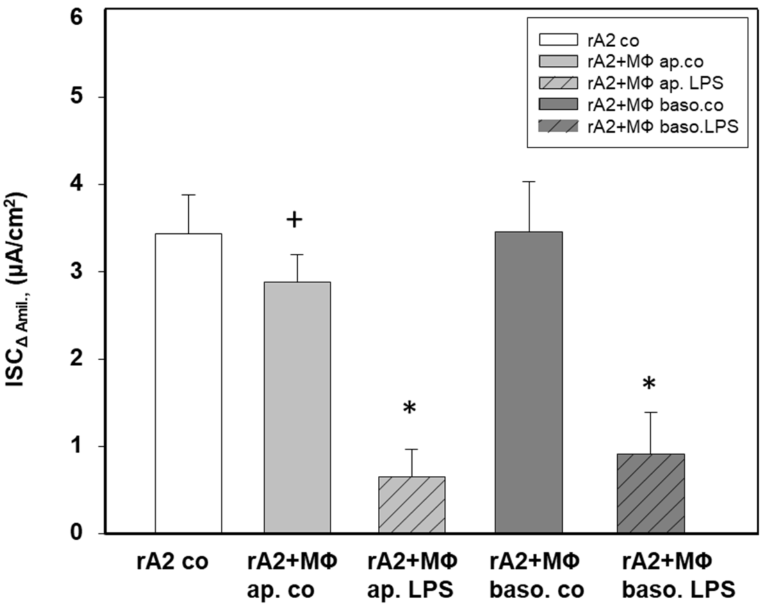

Figure S1A. Effects of LPS on $ISC_{\Delta amil}$ in apical and basolateral alveolar macrophages and rA2 co-cultures. Alveolar macrophages (MΦ) were co-cultured on the apical or basolateral side of rA2 and stimulated with LPS (1µg/ml) for 24h. Mean values ± SD of 6-8 independent cell preparations. * effect of LPS treatment on apical or basolateral side of the co-cultures (P<0.001), $^{+}$ effect of apical seeding MΦ on rA2 monolayers (p=0.018). MΦ: rat alveolar macrophages; rA2 co: rat alveolar epithelial cells alone, ap.: MΦ apically on rA2; baso.: MΦ basolaterally of rA2 cells.

Supplementary Figure S1B:

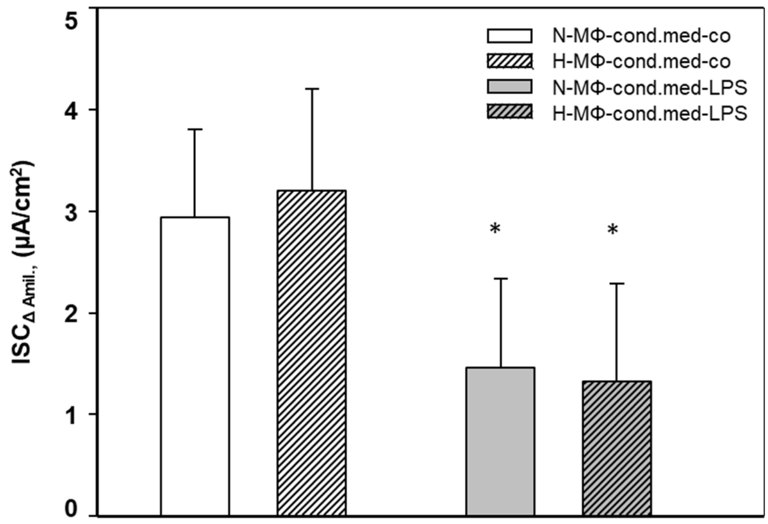

Figure S1B. Effects of normoxic or hypoxic LPS treated macrophage conditioned medium on $ISC_{\Delta amil}$ of normoxic rA2 mono-cultures. Alveolar macrophages (MΦ) were treated with LPS (1µg/ml) or not for 24h. Conditioned medium was kept on normoxic rA2 cells for 24h. Mean values ± SD of 7-8 independent cell preparations with at least 3 different conditioned media. * effect of LPS treatment (P<0.001), MΦ: rat alveolar macrophages; rA2: rat alveolar epithelial cells.

Supplementary Figure S2:

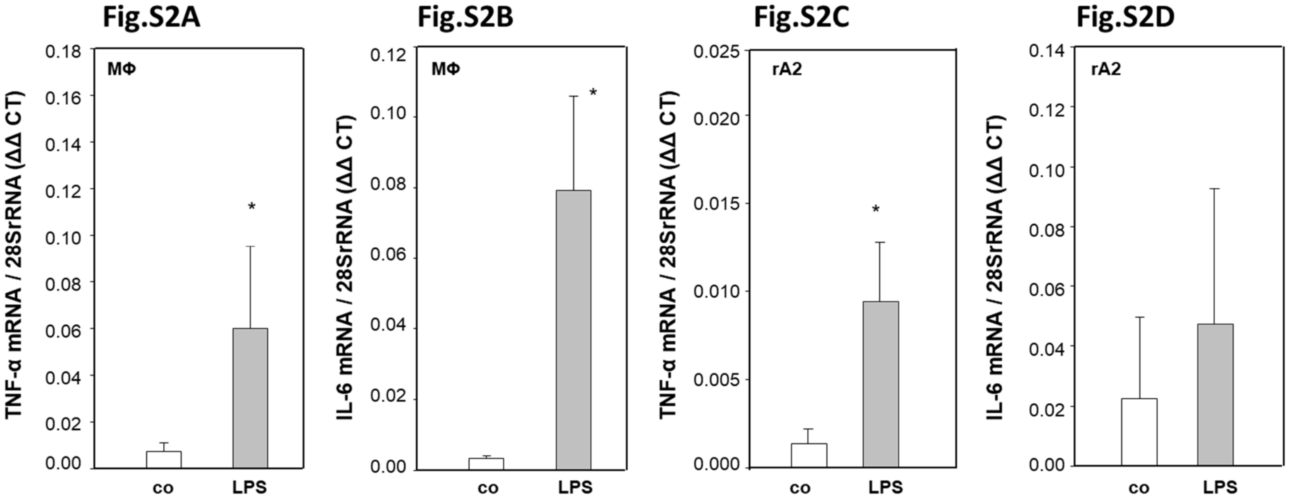

Figure S2. Effects of LPS on the mRNA expression of TNFα, IL-6 in alveolar macrophages and in rA2 cell mono-cultures. Cells were treated with LPS (1µg/ml) for 24hours. mRNA expression was normalized to 28S-rRNA. Mean values ± SD of 4-6 independent cell preparations. Level of significance was $P<0.05$: * effect LPS treatment compared to non-treated cells (t-test). MΦ: rat alveolar macrophages, rA2: rat alveolar epithelial cells

Supplementary Figure S3

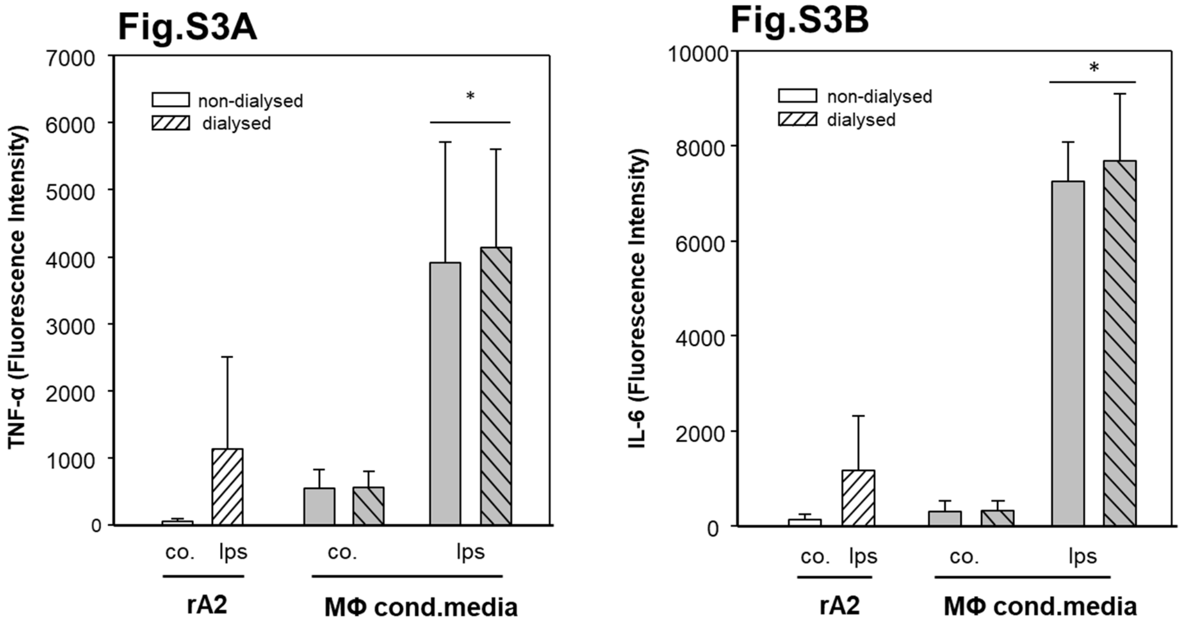

Figure S3. Effects of LPS on the levels of TNF-α and IL-6 in rA2 and alveolar macrophages. LPS (1µg/ml, 24h) treated rA2 cells and conditioned macrophage media were prepared and dialyzed as described in Method section. TNF-α (A) and IL-6 (B) from control and LPS treated macrophage medium (dialyzed and non-dialyzed) were quantified by the Luminex multiplex bead assay as described in Methods section. Cytokine levels are given as Fluorescence intensity (FI) which was acquired using Bio-Plex Manager software version 6.1 (Bio-Rad). Mean values ± SD of 4 independent preparations. Level of significance was $P<0.05$: * effect of LPS treatment. MΦ: rat alveolar macrophages, rA2: rat alveolar epithelial cells

Supplementary Figure S4

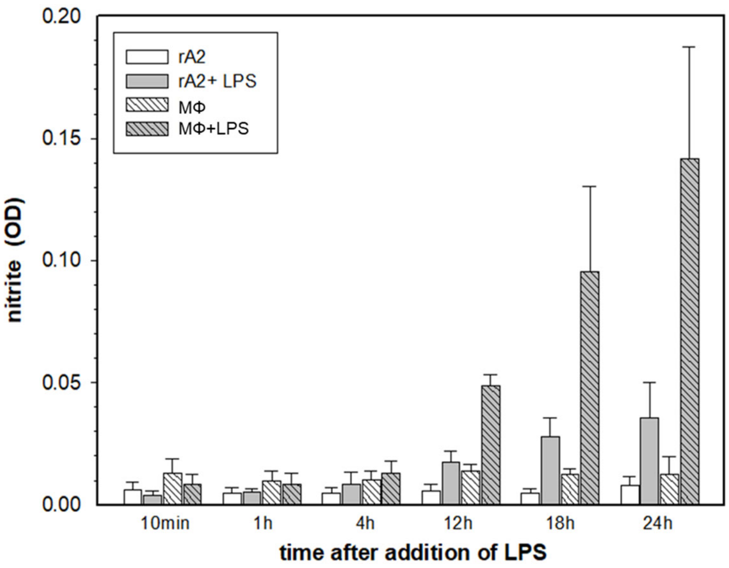

Figure S4. Effects of LPS on the accumulation of nitrite in culture medium of rA2 cells and alveolar macrophages in mono-cultures. rA2 and alveolar macrophages were stimulated with LPS (1µg/ml) for indicated time points. Culture media were removed, and nitrite product formation was measured with Griess reagent. Mean values ± SD of 2 independent preparations performed in triplicate experiments. MΦ: rat alveolar macrophages, rA2: rat alveolar epithelial cells

Supplementary Figure S5:

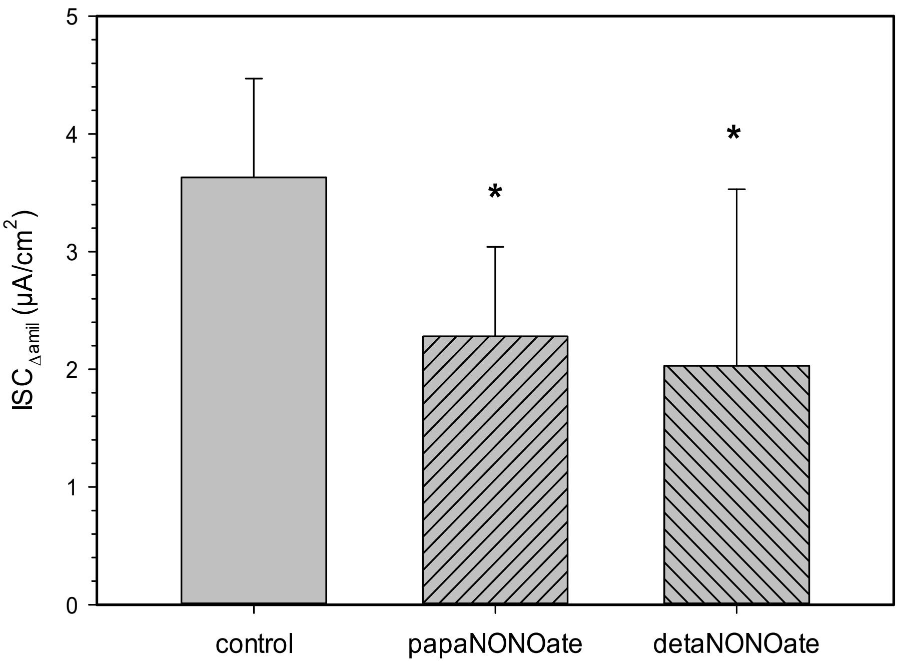

Figure S5. Effects of short and long term NO donors on ISCΔ$_{amil}$ of normoxic rA2 mono-cultures. Primary rA2 cell mono-layers incubated with short half-life NO-donor papaNONOate (50 µM) directly in the Ussing Chamber for 60 min or incubated for 24h with long half-life NO-donor detaNONOate (100 µM). Mean values ± SD of 7-10 experiments from at least 3 independent cell preparations. * effect of papaNONOate treatment (P=0.005), and effect of detaNONOate treatment (P=0.001).

Supplementary Figure S6:

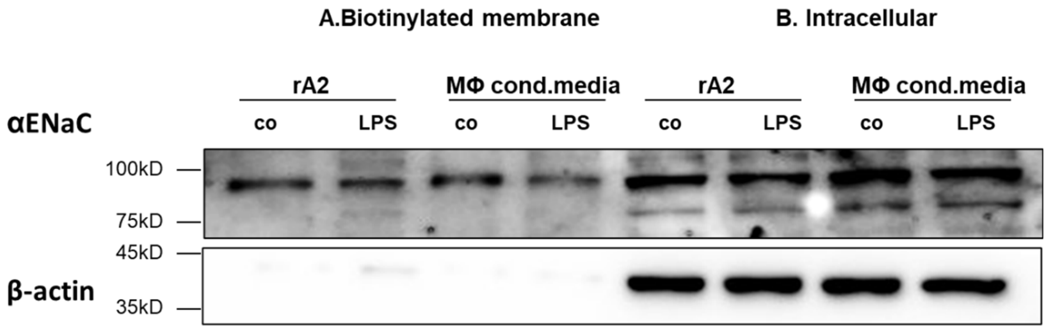

Figure S*6*. Plasma membrane and intracellular αENaC protein expression of rA2 cells treated with LPS stimulated macrophage conditioned media. Conditioned media from LPS stimulated macrophages were kept on primary rA2 cell monolayers for 24h. Biotinylated proteins represent the apical membrane fraction, non-biotinylated is considered intracellular. Representative immunoblots showing αENaC **(A)** surface expression (biotinylated). **(B)** Intracellular αENaC (non-biotinylated). β-actin was not detected in the surface (biotinylated) membrane fraction but only in the intracellular pool.

Supplementary Materials and Methods

Preparation of rat lung primary alveolar epithelial cells (rA2): Primary rA2 cells were isolated from lungs of normoxic male rats (Sprague-Dawley, 170-200g) as described earlier [1,2]. Experiments were approved by the Animal Protection Committee of the University of Heidelberg and by the Regierungspräsidium Karlsruhe, Germany (T-21/09; T-64/15). Briefly, rats were anesthetized (100 mg/kg Na-pentobarbital i.p., Trapanal, Byk Gulden, Germany) and lungs were perfused with a buffer composed of (in mM) 136 NaCl, 5.3 KCl, 5.6 glucose, 2.6 NaH_2PO_4, 10 HEPES, 0.15 EGTA, pH 7.4 at RT, while being ventilated. rA2 cells were isolated by elastase digestion (Elastin Products, USA), mincing of lung tissue, differential filtration, and differential adhesion in IgG-coated plates. Non-adherent cells were suspended in DMEM-N, i.e., DMEM supplemented with 10% neonatal calf serum, glutamine (4mM), and gentamycin (50µg/ml), and were plated on the side of the nucleopore filters facing the inner compartment at a density of 1.5 x 10^6 cells/cm². Both purity and viability of rA2 cells were >90%. Cells were cultured in normoxia (room air with 5% CO_2) in a liquid-liquid interface which allows collection of culture medium for analysis of released signaling molecules.

NR8383 rat alveolar macrophage cell line (NR8383): Because primary rat lung macrophages collected by bronco-alveolar lavage appeared to be in a stimulated state, experiments presented here were performed with the rat lung derived NR8383 alveolar macrophage cell line (ATCC, USA). Cells were grown in 75 cm^2 tissue culture flasks using DMEM-N (see above, rA2 cells). Both adherent and floating cells were harvested and mixed for further use between passage 6 and 12.

Co-culture of rA2 with NR8383: Two days after seeding rA2 cells had reached confluence indicated by a transepithelial electrical resistance (TEER) >250 Ohms x cm^2. In preliminary experiments, NR8383 macrophages were added to the apical or basolateral side of the transwells and were co-cultured for 24h before other treatments. Since results on ion transport and gene expression were similar with apical and basolateral placement of NR8383, and since no clean separation of cell types for RNA preparation was possible in the mixed culture, all further experiments were performed with the macrophages (3 x 10^5 cells in 1.5ml) added to the basolateral compartment.

Preparation of macrophage-conditioned media and dialysis: NR8383 grown in 75 cm^2 flasks for two days were treated (e.g., LPS, 1 µg/ml; L-NMMA, 1 mM, controls with respective solvent) for 24h in normoxia. Culture media were collected, non-adherent cells were removed by centrifugation, and media were stored frozen at -80°C until use with rA2 cell mono-cultures. To study possible effects of NO, nitrite and related products released from LPS stimulated macrophages, conditioned media from control and LPS-stimulated NR8383 was dialyzed using MW-500 Dalton cut-off dialysis tubing (Spectrum, Roth, Germany) against DMEM overnight and against fresh DMEM for another 4 hours. Dialyzed medium was sterile filtered and kept frozen at -80°C until use.

Treatments: rA2 cells reached confluency on day two after cell preparation. For rA2-NR8383 co-cultures; NR8383 were kept on the basolateral side of the rA2 cells for 24h. Next day NR8383 were treated with LPS (1 µg/ml) and L-NMMA (1 mM) (both from Sigma-Aldrich, Germany) and exposed to hypoxia (1.5% O_2; 5% CO_2, rest N_2) using an O_2/CO_2-controlled incubator (Binder, Germany) for up to 48 hours as indicated. Control cultures remained in normoxia. Experiments were also performed on rA2 mono-cultures with LPS (1 µg/ml) andL-NMMA (1mM).

Short and long half-life NO donor treatments: To study the effect of external applied NO on $ISC\Delta_{amil}$ of rA2 mono-layers were incubated with short (76min) NO donor papaNONOate (50 µM) directly in the Ussing Chamber for 60 min or incubated 24h with long half-life (~20h) NO-donor detaNONOate (100 µM). Electrophysiological measurements were performed as described in Methods section.

RNA isolation and quantitative RT-PCR: rA2 cells were washed once with phosphate buffered saline (PBS), macrophages were scraped off the surface, packed by centrifugation and washed with PBS. Cells were lyzed with RLT reagent (Qiagen, USA), and total RNA was isolated using the RNeasy micro kit (Qiagen, Germany) according to the manufacturer's instructions. RNA (0.1µg) was transcribed with Superscript II reverse transcriptase (Invitrogen, Life Technologies, Germany) using random hexamere primers (Roche, Germany). Real time quantitative PCR was performed in the LightCycler® (Model 480, Roche, Germany). The ABsolute QPCR SYBRgreen mix (Thermo Scientific, Germany) was used with QuantiTect®-primers (QuantiTect®, Qiagen, Germany) for the detection; 28S-rRNA was used as a housekeeping gene. The $2^{-\triangle\triangle}$Ct method was used to analyze the relative mRNA abundance[3].

Cytokine measurements:

Levels of TNF-α and IL6 in control and LPS stimulated macrophage conditioned medium (dialyzed and non-dialyzed) were quantified by the Luminex multiplex bead assay (Bio-Rad, Munich, Germany). Measurements were performed on a BioPlex200 System using the Bio-Plex Pro Cytokine Reagent Kit and Bio-Plex Pro Rat Cytokine sets (both Bio-Rad) according to manufacturer's instructions. Cytokine levels are given as Fluorescence intensity (FI) which was acquired using Bio-Plex Manager software version 6.1 (Bio-Rad).

Cell surface biotinylation and Western Blotting:

The surface expression of αENaC from LPS stimulated macrophage conditioned media was measured after biotinylation of apical membrane proteins [48,49]. Briefly, cells were washed with ice-cold PBS, incubated with NHS-SS-biotin (Pierce, Schwerte, Germany) for 20 min at 4°C, and washed with PBS containing glycine (100 mM). Cells were lyzed with lysis buffer (1% Triton X-100, 150mM NaCl, 5mM EDTA, 50mM Tris, pH 7.5), and lysates were centrifuged (2 min, 13.000 rpm, 4°C). Supernatants containing 200 µg of total protein were incubated with Streptavidin-agarose beads (Pierce, Schwerte, Germany) overnight at 4°C. Beads were pelleted by centrifugation. Supernatants (i.e. the non-biotinylated fraction) containing the intracellular proteins were collected and stored frozen. Biotinylated proteins representing the apical membrane fraction were eluted from the beads by heating at 95°C, 5 min, in 4x-sample buffer. Non-biotinylated and biotinylated samples were used for Western blotting. Proteins were separated on 10% SDS-PAGE and transferred onto PVDF membranes for Western blot analysis using the α-ENaC antibody (rabbit, PA1-920) was from Thermo Scientific, (Schwerte, Germany) and β-actin as reference. Secondary antibodies conjugated with horseradish peroxidase and enhanced chemiluminescence (GE Healthcare/Amersham, Germany) were used for detection. Band densities were measured using the Image J software (NIH, USA).

References:

1. Dobbs LG. Isolation and culture of alveolar type II cells. *Am J Physiol*. 1990;258:L134-L147. IN FILE. doi: PM:2185652

2. Mairbäurl H, Mayer K, Kim KJ, Borok Z, Bärtsch P, Crandall ED. Hypoxia decreases active Na transport across primary rat alveolar epithelial cell monolayers. *Am J Physiol*. 2002;282:L659-L665. IN FILE.
3. Rao X, Huang X, Zhou Z, Lin X. An improvement of the 2^(-delta delta CT) method for quantitative real-time polymerase chain reaction data analysis. *Biostat Bioinforma Biomath*. Aug 2013;3(3):71-85.
